# Metatranscriptomic Identification of Trubanaman Virus Sequences in Patient with Encephalitis, Australia

**DOI:** 10.3201/eid3112.251190

**Published:** 2025-12

**Authors:** Krispin Hajkowicz, John Woodford, Elango Subramonia Pillai, Andrea Henden, Kym Lowry, Mary E. Petrone, Patrick N.A. Harris, Edward C. Holmes

**Affiliations:** Royal Brisbane and Women’s Hospital, Brisbane, Queensland, Australia (K. Hajkowicz, E. Subramonia Pillai, A. Henden, P.N.A. Harris); University of Queensland Centre for Clinical Research, Brisbane (K. Hajkowicz, K. Lowry, P.N.A. Harris); The School of Medical Sciences, University of Sydney, Sydney, New South Wales, Australia (K. Hajkowicz, M.E. Petrone, E.C. Holmes); Ipswich Hospital, Ipswich, Queensland, Australia (J. Woodford); Queensland Institute of Medical Research-Berghofer, Brisbane (J. Woodford, A. Henden); Queensland Paediatric Infectious Diseases Sakzewski Laboratory, Brisbane (K. Lowry); Pathology Queensland, Brisbane (P.N.A. Harris)

## Abstract

Using metatranscriptomics, we identified Trubanaman virus in cerebrospinal fluid from a severely immunocompromised man who died of encephalitis in Queensland, Australia. Virus sequences were related to orthobunyaviruses previously detected in mosquitoes in Australia. Testing for other causes yielded negative results, suggesting that Trubanaman virus was the cause of this fatal encephalitis case.

Approximately 50% of global encephalitis cases remain undiagnosed by conventional testing (*1*). Metagenomic next-generation sequencing (mNGS), particularly metatranscriptomics (i.e., total RNA sequencing), is an emerging approach to infection diagnosis that reveals all nucleic acid in a sample, making it ideal for detecting novel and emerging pathogens (*2*).

*Orthobunyavirus* (order Bunyavirales) is a diverse genus of negative-sense single-stranded RNA viruses recognized to cause febrile illness and encephalitis in humans globally (*3*). The best described orthobunyaviruses are La Crosse virus and Jamestown Canyon virus, both of which rarely cause encephalitis, permanent neurologic sequalae, or death (*4**,**5*). Jamestown Canyon virus is associated with meningoencephalitis in immunocompromised persons (*5*), whereas the emerging Oropuche virus is associated with fever, headache, myalgias, and rare cases of meningoencephalitis and has recently expanded its range in Central and South America (*6*). We used metatranscriptomics to investigate a case of encephalitis in an immunocompromised person in Australia.

The study was approved by the Metro-North Health Human Research Ethics Committee and written informed consent was obtained from the patient and his next of kin. Metatranscriptomic sequencing and analysis methods are detailed (Appendix).

A man in his 50s who lived in West Moreton, Queensland, Australia, was admitted for a volunteer unrelated donor allogeneic hemopoietic stem cell transplantation with posttransplant cyclophosphamide and tacrolimus for B-cell acute lymphoblastic leukemia in complete remission one. There was no central nervous system involvement. He received 8 cycles of rituximab-hyper cyclophosphamide, vincristine, doxorubicin, and dexamethasone before transplantation. The transplant was complicated by a polymicrobial bloodstream infection that was successfully treated with intravenous daptomycin, as well as mucositis and diarrhea.

On day 18 after the hemopoietic stem cell transplantation, the patient experienced fever to 38.8°C, tachycardia to 10^9^ beats/min, muscular pain, intermittent headache, and confusion manifesting as slow and tangential answers to questions, difficulty word-finding, reduced oral intake, disorientation to time and place, and delusions such as thinking that he had been in a car accident. The onset coincided with recovery of his neutrophil and lymphocyte count. His confusion fluctuated but generally deteriorated. Twenty-two days later, a cerebrospinal fluid (CSF) examination was performed (Table). Magnetic resonance imaging (MRI) of the brain was also performed, and results were unremarkable. However, results of an electroencephalograph were abnormal, showing mild, diffuse cortical dysfunction but no epileptiform activity. Results of a nasopharyngeal nucleic acid amplification test (NAAT) were positive for rhinovirus. Stool, blood, and urine culture and NAAT results were negative for viruses, bacteria, and fungi (Table). He was unresponsive to corticosteroids, and during the next few months, his level of consciousness, function, and speech declined; serial MRIs showed progressive cerebral atrophy. He died 6 months after the onset of confusion.

**Table:** Results of cerebrospinal fluid testing in an immunocompromised patient with encephalitis in study of metatranscriptomic identification of Trubanaman virus, Australia*

Test	Result
Leukocytes, × 10^6^ cells/L (reference range <5)	2
Erythrocytes, × 10^6^ cells/L (reference range <5)	61
Protein, mg/L (reference range 150–500)	570
Glucose, mmol/L (reference range 2.2–3.9)	5.3
Microscopy and culture	No bacteria or fungi detected
NAAT for herpes simplex 1 and 2, cytomegalovirus, varicella zoster virus, human herpesvirus 6, John Cunningham virus, enteroviruses, and parechoviruses	Negative
NAAT for *Escherichia coli*, *Haemophilus influenzae*, *Listeria monocytogenes*, *Neisseria meningitidis*, *Streptococcus agalactiae*, *Streptococcus pneumoniae*, and *Cryptococcus* spp.	Negative
Cryptococcal antigen in cerebrospinal fluid	Negative
Neuronal antibodies Hu, Ri, Yo, and Purkinje cell cytoplasmic types 2 and Tr	Negative
N-methyl-D-aspartate, GABA-B, and AMPA receptors	Negative
CV2/collapsin response mediator protein 5	Negative
Ma/Ta, glutamic acid decarboxylase, and amphyphysin antibodies	Negative

*NAAT, nucleic acid amplification test.

Metatranscriptomic sequencing of the patient’s CSF using NovaSeq (Illumina, https://www.illumina.com) generated a total of 57,452,775 paired reads, of which 74 matched the M glycoprotein precursor of Trubanaman, Murrumbidgee, and Buffalo Creek viruses (i.e., the Mapputta group, which likely represents a single species within the genus *Orthobunyavir*us). E-values were <10^−116^. From some of those reads, we assembled a single contig of 270 bp (GenBank accession no. PV702715), denoted Trubanaman virus West Moreton (Figure). We did not recover reads from the RNA-dependent RNA polymerase (RdRp) or other virus genes. The water control was negative for bunyaviruses. Similarly, metatranscriptomic analysis was negative for other known or putative human pathogenic viruses, bacteria, fungi, and parasites, and no other candidate pathogens were identified.

**Figure:**
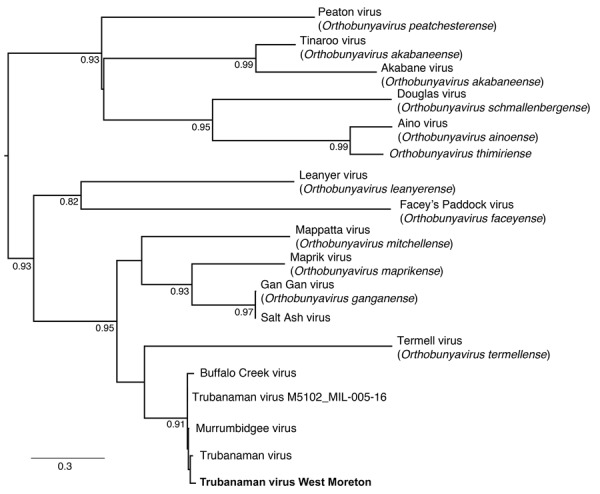
Phylogenetic tree of orthobunyavirus M segment sequences from this study and previously collected mosquito samples in study of metatranscriptomic identification of Trubanaman virus in patient with encephalitis, Australia. Bold font indicates the human sequence from this study; other sequences are from mosquito orthobunyaviruses previously identified in Australia. Sequences were aligned using MAFFT (https://mafft.cbrc.jp/alignment/server/index.html). The phylogeny was estimated using the maximum-likelihood approach in PhyML (http://atgc.lirmm.fr/phyml), by using the general time reversible model of nucleotide substitution and gamma-distributed rate variation among sites. Bootstrap support values are displayed at nodes. Scale bar indicates nucleotide substitutions per site.

Using metatranscriptomics, we identified Trubanaman virus sequences in a CSF sample from a person with encephalitis. Extensive testing for other infectious, autoimmune, and malignant causes yielded negative results. In the context of a high-risk immunocompromised person with typical clinical manifestations of encephalitis, our findings support, but do not confirm, that Trubanaman virus was the cause of the patient’s encephalitis. PCR could not be performed on the original sample because it was fully depleted for conventional testing and sequencing, although no viable routes to sample contamination existed. Follow-up testing of CSF collected 6 weeks later was negative by both metatranscriptomics and orthobunyavirus-specific PCRs targeting the N protein and RdRp.

Trubanaman and related viruses have been detected in mosquito populations throughout Australia (*7*). Patients with a suspected arthropodborne virus infection in New South Wales exhibited neutralizing antibody prevalences of 4.7% to Gan Gan virus (GGV) and 1.4% to Trubanaman virus (*8*). GGV was associated with an acute febrile illness and polyarthritis in 3 persons in Australia who had significant titer rises in paired serum samples, as well as GGV-specific IgM (*9*). In addition, serologic evidence suggests that kangaroos, feral animals, and domestic horses are reservoirs for orthobunyaviruses in Australia (*9*). Of note, 2 bunyavirus-associated cases of fatal meningoencephalitis in immunocompromised persons were recently described in the United States using CSF mNGS (*10*). Further research is required to establish the pathogenic role of Trubanaman virus as a cause of encephalitis in Australia and to determine the arthropod vectors, zoonotic reservoirs, and seroprevalence. However, our findings suggest that Trubanaman virus was the cause of this fatal encephalitis case, and clinicians should be aware of the possibility of infection with this virus in similar cases.

Appendix Additional information for metatranscriptomic identification of Trubanaman virus in patient with encephalitis, Australia.
