# Supplementary material for: Metatranscriptomic Identification of Trubanaman Virus Sequences in Patient with Encephalitis, Australia
Source: Emerg Infect Dis. 2025 Dec;31(12):2332–5. doi: 10.3201/eid3112.251190 (PMC12782261; doi:10.3201/eid3112.251190)
Supplement: Appendix — Additional information for metatranscriptomic identification of Trubanaman virus in patient with encephalitis, Australia. [file 25-1190-Techapp-s1.pdf]

Article DOI: https://doi.org/10.3201/eid3112.251190

EID cannot ensure accessibility for supplementary materials supplied by authors. Readers who have difficulty accessing supplementary content should contact the authors for assistance.

Metatranscriptomic Identification of Trubanaman Virus in Patient with Encephalitis, Australia

Appendix

Methodology

Written informed consent was obtained initially from the patient and additionally from his next of kin. A CSF sample was accessed after completion of routine testing. RNA was extracted using the Qiagen RNeasy® Plus Mini Kit using the QiaCUBE (Qiagen, Australia), followed by library preparation with the Illumina Ribo-Zero® Plus rRNA Depletion Kit (Illumina, San Diego, CA, USA). Total RNA sequencing was performed on the Illumina Novaseq™ X Plus, using a 10B lane with 21 multiplexed samples and one RNAse free water control. After trimming and deduplication with Fastp 0.22.0 (*1*) and filtering low complexity reads with Prinseq 0.20.4 (*2*), human reads were removed using Bowtie2 2.4.4 (*3*) and Kraken2 2.0.8-beta (*4*,*5*), followed by rRNA removal with Sortmerna 4.3.3 (*6*). Contigs were assembled using Megahit 1.1.3 (*7*) prior to BLAST nt (Blast+ 2.11.0) (*8*) and nr (Diamond 2.0.11) (*9*) using broad microbial databases. Additional contig assembly, sequence alignment and BLAST analysis was performed with Chan-Zukerberg (CZ) ID (*10*). A phylogenetic tree comparing this contig with other Australian orthobunyaviruses on the NCBI virus database was estimated using MAFFT 7.49 (*11*) sequence alignment and phylogenetic analysis employing the maximum likelihood method available in PhyML (*12*), employing the General Time Reversible model of nucleotide substitution with a gamma distribution of among-site rate variation.

The contig nucleotide sequence of Trubanaman virus determined here has been deposited on NCBI/GenBank under accession number PV702715.

Results of the BLAST analysis

The E-value E()- represents the expected number of random alignments that would score as well or better than the observed alignment purely by chance. An e-value $<10^{-10}$ suggests strong sequence homology and that an alignment is unlikely to occur by chance (*13*).

Significance thresholds (Appendix Table):

>10x reads compared to RNA-free water control

≥10 reads

Not a bacteriophage, environmental picorna-like virus or known contaminant

References

1. Chen S, Zhou Y, Chen Y, Gu J. fastp: an ultra-fast all-in-one FASTQ preprocessor. Bioinformatics. 2018;34:i884–90. https://doi.org/10.1093/bioinformatics/bty560

2. Schmieder R, Edwards R. Quality control and preprocessing of metagenomic datasets. Bioinformatics. 2011;27:863–4. https://doi.org/10.1093/bioinformatics/btr026

3. Langmead B, Salzberg SL. Fast gapped-read alignment with Bowtie 2. Nat Methods. 2012;9:357–9. https://doi.org/10.1038/nmeth.1923

4. Wood DE, Lu J, Langmead B. Improved metagenomic analysis with Kraken 2. Genome Biol. 2019;20:257. https://doi.org/10.1186/s13059-019-1891-0

5. Hall MB, Coin LJM. Pangenome databases improve host removal and cyanobacteria classification from clinical metagenomic data. Gigascience. 2024;13:gia010. https://doi.org/10.1093/gigascience/gia010

6. Kopylova E, Noé L, Touzet H. SortMeRNA: fast and accurate filtering of ribosomal RNAs in metatranscriptomic data. Bioinformatics. 2012;28:3211–7. https://doi.org/10.1093/bioinformatics/bts611

7. Li D, Liu CM, Luo R, Sadakane K, Lam TW. MEGAHIT: an ultra-fast single-node solution for large and complex metagenomics assembly via succinct de Bruijn graph. Bioinformatics. 2015;31:1674–6. https://doi.org/10.1093/bioinformatics/btv033

8. Camacho C, Coulouris G, Avagyan V, Ma N, Papadopoulos J, Bealer K, et al. BLAST+: architecture and applications. BMC Bioinformatics. 2009;10:421. https://doi.org/10.1186/1471-2105-10-421

9. Buchfink B, Reuter K, Drost HG. Sensitive protein alignments at tree-of-life scale using DIAMOND. Nat Methods. 2021;18:366–8. https://doi.org/10.1038/s41592-021-01101-x

10. Kalantar KL, Carvalho T, de Bourcy CFA, Dimitrov B, Dingle G, Egger R, et al. IDseq: an open-source cloud-based pipeline and analysis service for metagenomic pathogen detection and monitoring. Gigascience. 2020;9:giaa111. https://doi.org/10.1093/gigascience/giaa111

11. Katoh K, Standley DM. MAFFT multiple sequence alignment software version 7: improvements in performance and usability. Mol Biol Evol. 2013;30:772–80. https://doi.org/10.1093/molbev/mst010

12. Guindon S, Dufayard JF, Lefort V, Anisimova M, Hordijk W, Gascuel O. New algorithms and methods to estimate maximum-likelihood phylogenies: assessing the performance of PhyML 3.0. Syst Biol. 2010;59:307–21. https://doi.org/10.1093/sysbio/syq010

13. Pearson WR. An introduction to sequence similarity ("homology") searching. Curr Protoc Bioinformatics. 2013;42:3.1.1–8.

Appendix Table. Other microorganisms detected above significance thresholds

Pathogen	Reads per million	Reads	Contigs	E-value	Comments
Bacteria					
Halothiobacillus neapolitanus	1889.5	12251	1	10^{-308}	Likely to be a reagent contaminant
Staphylococcus epidermidis	697	4521	20	10^{-94}	Likely to be a skin contaminant introduced during lumbar puncture. Other coagulase-negative Staphylococci commonly associated with skin contamination also present.
Halothiobacillus neapolitanus	1889.5	12251	1	10^{-308}	Likely to be a reagent contaminant
Corynebacterium tuberculostearicum	151	979	2	10^{-76}	Likely to represent a skin contaminant introduced at the time of lumbar puncture. Other Corynebacteria commonly associated with skin contamination were also detected.
Amycolaopsis methanolica	388	2519	2	10^{-84}	Likely to represent a contaminant, not associated with human disease.
Moraxella osloensis	394	2558	5	10^{-84}	Was also present in the water control (25.6 rpm). Reads covered only 0.2% of the M. osloensis genome. M. osloensis is a common laboratory contaminant.
Fungi					
Malassezia restricta	2823	18307	57	10^{-95}	Also present in the water control. Likely to represent reagent contamination.
